# An Encyclical from Charing Cross

**Published:** 1921-08-20

**Authors:** 


### An Encyclical from Charing Cross.

### HOW TO INTEREST HOSPITAL SUPPORTERS.

Mr. George Verity, the Chairman of Charing Cross Hospital, is accustomed each year to issue a sort of encyclical letter from the hospital to his subscribers. In 1921 he is no less enthusiastic and vigorous than usual. He points out with pride that the financial recovery of Charing Cross Hospital in the past few years, which he emphasised in *The Times*, did much to allay the general panic and to show the Government and the people generally that the voluntary system was very far from exhausted. He believes, in fact, that the witness of Charing Cross Hospital did much to save the voluntary system from the State; and this part of his letter will hearten the hospital's supporters still further. He records that in the first year since patients' contributions were introduced over £8,000 has been received—an initial sum that provides "almost 25 per cent. of the cost of running the hospital." The success of these contributions is attributed to the work of Mrs. Pillow, the lady almoner, and to the sympathetic staff which has been formed.

Mr. Verity thinks that injudicious capital expenditure has led many hospitals into difficulties, and he therefore confined last year's outlay to the renovation of the nurses' home and recreation-rooms; the re-painting of the façade, wards, corridors, and theatres; the extension and re-equipment of the *x*-ray department; the enlargement of the lady almoner's office; and to overhauling the electric light, bells, and fire alarms. Under this policy the works postponed become of equal importance: more rooms for nurses, a lift for the nurses' home, a freezing apparatus, and a hospital laundry. Mr. Verity is opposed to a hospital laundry as troublesome and expensive. He adds that the hospital's programme was "comfortably carried out on the year's income," and that the hospital can profess: no debts, no overdraft, no complaints that it is not supported. This attitude, we read, so much delighted one person that he has become an anonymous donor of £500; and some Englishmen in Shanghai have sent £12,000 to endow a ward.

An interesting point is made in regard to the convalescents. The service is maintained and extended "at half the cost it used to be when we were encumbered with a millstone round our necks in the shape of a Home." The hospital's supporters are also informed that there is no shortage of applicants for the nursing staff at Charing Cross Hospital.

It cannot be denied that this "review of the year" is likely to achieve its object and inspirit those to whom it is addressed. It has the merits and defects of an extremely personal statement. Those who read it will feel that the hospital has a policy, that it is the policy in which the Chairman completely believes, and that, if they will support him, he will succeed in carrying it out. The consistent notes of personal cheerfulness and assurance are a change from the appealing cries for aid to which the public is accustomed. It is also fair to say that Mr. Verity's statement of the facts, relating, each of them, to the policy to which he is committed, makes them more interesting than any ordinary survey. If one may venture a criticism, it is that coloured inks, personal pronouns and underlines can be overdone. But, despite these, the survey is alive, and Mr. Verity's convictions on such subjects as hospital laundries, convalescent homes, and capital expenditure undoubtedly make the reader interested in questions which, as ordinarily presented, do not seem to invite personal thought or animated discussion.

